# Matching methods to create paired survival data based on an exposure occurring over time: a simulation study with application to breast cancer

**DOI:** 10.1186/1471-2288-14-83

**Published:** 2014-06-26

**Authors:** Alexia Savignoni, Caroline Giard, Pascale Tubert-Bitter, Yann De Rycke

**Affiliations:** 1 Service de Biostatistique, Institut Curie, 26 rue d’Ulm, 75005 Paris, France; 2 Inserm, CESP Centre for research in Epidemiology and Population Health, U1018, Biostatistics Team, F-94807 Villejuif, France; 3 Univ Paris-Sud, UMRS1018, F-94807 Villejuif, France; 4 Institut Curie, Pharmacological Unit, Saint-Cloud, France; 5 Institut Curie, Public Health Team, Paris, France

**Keywords:** Matching on time-dependent covariates, Matched time-to-event data, Correlated survival data, Event occurring over time, Stratified Cox model, Marginal Cox model, Pregnancy, Breast cancer, Simulation study

## Abstract

**Background:**

Paired survival data are often used in clinical research to assess the prognostic effect of an exposure. Matching generates correlated censored data expecting that the paired subjects just differ from the exposure. Creating pairs when the exposure is an event occurring over time could be tricky. We applied a commonly used method, Method 1, which creates pairs *a posteriori* and propose an alternative method, Method 2, which creates pairs in “real-time”. We used two semi-parametric models devoted to correlated censored data to estimate the average effect of the exposure $\bar{\text{HR}}(t)$: the Holt and Prentice (*HP*), and the Lee Wei and Amato (*LWA*) models. Contrary to the *HP*, the *LWA* allowed adjustment for the matching covariates (*LWA*_*a*_) and for an interaction (*LWA*_*i*_) between exposure and covariates (assimilated to prognostic profiles). The aim of our study was to compare the performances of each model according to the two matching methods.

**Methods:**

Extensive simulations were conducted. We simulated cohort data sets on which we applied the two matching methods, the *HP* and the *LWA*. We used our conclusions to assess the prognostic effect of subsequent pregnancy after treatment for breast cancer in a female cohort treated and followed up in eight french hospitals.

**Results:**

In terms of bias and *RMSE*, Method 2 performed better than Method 1 in designing the pairs, and *LWA*_*a*_ was the best model for all the situations except when there was an interaction between exposure and covariates, for which *LWA*_*i*_ was more appropriate. On our real data set, we found opposite effects of pregnancy according to the six prognostic profiles, but none were statistically significant. We probably lacked statistical power or reached the limits of our approach. The pairs’ censoring options chosen for combination Method 2 - *LWA* had to be compared with others.

**Conclusions:**

Correlated censored data designing by Method 2 seemed to be the most pertinent method to create pairs, when the criterion, which characterized the pair, was an exposure occurring over time. In such a setting, the *LWA* was the most appropriate model.

## Background

Five percents of breast cancers occur among women before the age of 40 and in fewer than 2% of women under 35 [1]. Physicians are more and more faced with issues of post-treatment pregnancy, such as the optimal time before considering conception. In physiopathological terms, a pregnancy after breast cancer is not advisable, especially in patients with positive hormonal receptors [2,3]. However, many observational studies report that pregnancy does not have any impact on the evolution of breast cancer [4]-[6], and could even have a long-term protective effect [2,6]-[10]. This phenomenon can be due to the so-called “healthy mother bias” [7], *i.e.* only women who are in good health will undertake a pregnancy after treatment for breast cancer.

Pregnancy is therefore related to the prognostic condition of the patient, and its occurrence might change the prognostic effect of some factors, for instance, the hormonal receptors. The prognosis of positive hormonal receptors could be qualitatively (interaction) different before and after pregnancy (*i.e.* interaction between hormonal receptor and pregnancy).

In an attempt to control for this “healthy mother effect”, various statistical approaches have been used. Some authors used the standard Cox model [11] with pregnancy considered as a covariate whose value depends on time, and adjusting for the known prognostic factors at the diagnosis of cancer, reflecting the gravity of the disease [2,6,8,9]. In spite of this adjustment for disease grade at diagnosis, pregnancy still remained a significant good prognostic factor, indicating a long-term protective effect of pregnancy, which is difficult to explain and to interpret. Others tackled the problem from the angle of an “illness-death” model [12]-[14] which makes it possible to describe the natural history of the disease, taking into account the prognostic profiles of the patients who had or did not have a pregnancy [15]. This model provided better understanding and interpretation of the effect of pregnancy by comparing transition probabilities to relapse between women who had or did not have a pregnancy. Moreover, taking into account a possible interaction between prognostic profiles and the pregnancy improved the estimation of the pregnancy prognostic effect. Overall prognosis was not adversely affected by subsequent pregnancy. However, to allow adjustment, such a complex model requires enough pregnancies and events of interest.

In both previous approaches, the adjustment relied only on known prognostic factors. However, measured prognostic factors might not be enough to characterize the prognostic status in such a disease. Matching subjects designs might be helpful to accomplish that.

Other researchers have conducted paired studies: pregnant and non-pregnant women were matched on the main known prognostic factors (hormonal receptor, proliferation level, nodal involvement, use of chemotherapy, year of diagnosis), and the non-pregnant had to be disease-free for as long as the time from diagnosis to pregnancy of the pregnant women [3]-[5,7,10]. By this matching carried out on known and measured factors, one can suppose that the subjects of the same pair also share non-observable, not observed or not measured factors, in addition to the factors of pairing. Thus, this design may improve the control of the “healthy mother effect” compared to the two approaches presented above.

However, to our knowledge, in such a case and contrary to the first methods cited previously, researchers [3]-[5,7,10] did not take into account the fact that pregnancy was an event occurring over time. They matched the pregnant woman to a non-pregnant one *a posteriori*, *i.e.* at the end of the follow up study, knowing which women were pregnant and which were not over the study period. They analyzed the data as if these pairs were *a priori* known and created at diagnosis, *i.e.* at time *t*=0. Moreover, they always used the stratified Holt and Prentice semi-parametric model (*HP*) [16] to estimate the pregnancy prognostic effect, whereas other semi-parametric models devoted to censored correlated data are available such as frailty models [17,18] and marginal models [19]-[22]. Frailty models model the time distribution conditionally to a random effect (frailty covariate), specific to each pair, and which is not observed. The structure of correlation has to be defined. The latter leave the nature of dependence among paired failure times completely unspecified.

Non-parametric [23,24] and parametric [25] approaches have been developed, but we focus on the semi-parametric approach, more specifically on the commonly used marginal semi-parametric model. This marginal approach was developed by Wei, Lin and Weissfeld [19] to analyze subjects with multiple events, and then Lee, Wei and Amato [20] adapted it to clustered subjects.

In this paper, we use the marginal paired proportional hazards model of Lee, Wei and Amato (*LWA*) [20] and the Holt and Prentice stratified model [16] (*HP*). The main difference between them lies in the ability of the *LWA*[20] model to adjust for matching covariates and for the possible interaction between the covariate and the exposure, contrary to the *HP* model. Mehrotra *et al.*[26] proposed an efficient alternative to the stratified Cox model analysis to estimate the exposure effect, which does not require the assumption of a common hazard ratio across strata. However, that model is not adapted to our particular context of a large number of strata, with very small sample size per stratum (in our work, a stratum is a pair), thus it will not be studied here.

With *HP* and *LWA* models, we considered two different methods to create our pairs: the *a posteriori* one commonly used and described previously, and a new one designing the pairs “in real-time” by taking into account the occurrence of the event over time, *i.e.* the pregnancy, which characterizes the subject’s group within the pair.

The goal was to determine the combination between matching methods (*a posteriori* and in “real-time”) and models (*HP* and *LWA*), which is the most efficient in terms of bias and Root Mean Square Error (*RMSE*) to estimate and test the pregnancy prognostic effect. In the following, pregnancy is referred to as the “exposure” occurring over time and the pairs are composed of an exposed and a non-exposed subject.

In the next section, the two matching methods as well as the models of analysis are presented. In Section ‘Simulations’, extensive simulations are used to analyze the performance of these Method - Model combinations in term of bias and *RMSE* of the estimated effect related to the exposure occurring over time. In Section ‘Real data application’, our findings are applied to real data in order to analyze the effect of a subsequent pregnancy on breast cancer evolution [9]. A brief discussion concludes the paper.

## Methods

The subjects are matched on all the known prognostic factors represented by vector **Z**. In the following, index *j* corresponds to the rank of the exposed subject occurring at the *j*th position’s order among the *n*_*e*_ exposed subjects at the end of the study (*j*=1,…,*n*_*e*_), and index *i* corresponds to the subject among the *n* subjects of the study (*i*=1,…,*n*). Let *t*_*i*_ being defined as the follow-up time of each subject *i*, and *t*_*E* *i*_ as the time exposure for a subject *i*; for a subject who is never exposed, we consider $t_{\text{Ei}}=+\infty$. $R_{m}\left(t\right)$ represents the subjects at risk of event and non-exposed at time *t* for the method *m* (*m*=1 or 2). Let the set of paired subjects being defined as *P*_*j*_={*j*,*i*(*j*)}, with *i*(*j*) the subject *i* of $R_{m}\left(t_{E_{j}}\right)$ chosen for the *j*th exposed subject.

### The two matching methods

The two matching methods applied in the context where exposure is an event occurring over time, are presented.

Method 1 matches the subjects as follows: all the subjects *i* exposed ($t_{\text{Ei}}<+\infty$) are matched with a subject who was not exposed during the whole period of follow-up. This method is called an *a posteriori* method because the groups exposed and non-exposed are defined at the end of the study and are considered to be known and created at time *t*=0. According to Method 1, if a subject *j* undergoes an exposure at time *t*_*E* *j*_, then the eligible subject’s set $R_{1}\left(t_{\text{Ej}}\right)$ of subjects *i* eligible to be matched to *j* could be written as follows: $R_{1}\left(t_{\text{Ej}}\right)=\left\{i\neq j/t_{i}\geq t_{\text{Ej}}\text{AND}t_{\text{Ei}}=+\infty \right\}$. This approach has been commonly used in the literature [3]-[5,7,10] with exposure not being considered as time-dependent.

As exposure is a time-dependent event, a second approach is proposed which designs the pairs “in real-time”: according to Method 2, if a subject *j* undergoes an exposure at time *t*_*E* *j*_, then the eligible subject’s set $R_{2}\left(t_{\text{Ej}}\right)$ of subjects *i* eligible to be matched to *j* at time *t*_*E* *j*_, could be written as follows: $R_{2}\left(t_{\text{Ej}}\right)=\{i\neq j/t_{i}\geq t_{\text{Ej}}$ AND *t*_*E* *i*_>*t*_*E* *j*_}. $R_{2}\left(t_{\text{Ej}}\right)$ includes subjects at risk that are not yet exposed at *t*_*E* *j*_ and will be exposed after, and subjects who will never be exposed. A pair composed of an exposed subject and a non-exposed one who would never undergo the exposure is called a “perfect pair”, whereas it would be an “imperfect pair”. As a result, the non-exposed subject of an imperfect pair could be matched after exposure with another non-exposed subject.

Method 2 is similar to the one proposed by Lu *et al.* in case-cohort studies [22] where the membership in the exposed and unexposed groups is the outcome to be explained, whereas in our work it is an explanatory variable.

For both methods, exposed and not exposed subjects are matched according to the covariate vector **Z**, and the not exposed subject has to be disease-free for as long as the time from the starting point (disease diagnosis) to the exposure time.

For both methods, if several non-exposed subjects can be matched with an exposed one, the matched non-exposed subject is randomly chosen from the set of eligible subjects $R_{m}\left(t\right)$; if no non-exposed subjects are available, the exposed subject cannot be paired and is thus excluded from the analysis.

Note that even if a subject could belong to two different pairs with Method 2, these two pairs are independent while they are never at risk at the same time *t*.

### The statistical models

In the following, *λ*_*i*_(*t*) is the instantaneous hazard function of outcome to be estimated for pair *P*_*j*_. It is noted $\lambda_{i}\left(t,Z_{i}\right)$ to specify that the estimation is made on the pair *P*_*j*_, which is composed of the exposed subject *j* and the non-exposed subject *i* matching on **Z**_*i*_. This notation is the same for all the models studied, even those where the adjustment for **Z**_*i*_ is not available.

For all the models presented below, $E_{i}\left(t\right)$ corresponds to the time-dependent exposure status and is defined as follows: $E_{i}\left(t\right)=0$ if *t*<*t*_*E* *i*_, and *E*_*i*_(*t*)=1 if *t*≥*t*_*E* *i*_. The pair of subjects is also defined by a time-dependent covariate: $P_{i}\left(t\right)=j$ if *i*∈*P*_*j*_ and *t*∈[*t*_*E* *j*_;*t*_*i*_], or $P_{i}\left(t\right)=0$ otherwise.

#### Holt and Prentice stratified Cox model

Holt and Prentice [16] adapted the standard Cox model [11] to analyze matched paired data.

The instantaneous hazard function is written for each subject *i* as

$$\lambda_{i}\left(t,Z_{i}\right)=\lambda_{0i(j)}\left(t\right)\exp\left(\gamma \left(t\right)E_{i}\left(t\right)\right)\;\text{(HP)}$$

$\lambda_{0i(j)}\left(t\right)$ is a pair-specific baseline hazard function that is assumed to be identical for both subjects of pair *P*_*j*_, considered here as strata; it is considered as a nuisance parameter not to be estimated. The exposure effect $\exp\left(\gamma \left(t\right)\right)$ is then estimated, considering the between-pair heterogeneity, by allowing the instantaneous baseline hazard to be different within each pair. It is assumed to be identical across strata (no interaction between the exposure and the pairs) and thus to be implicitly common for the whole exposed population: $\exp\left(\gamma \left(t\right)\right)$ is defined as the population-weighted average of the stratum-specific hazard ratios. However, if this assumption is incorrect, *i.e.* in the presence of a true (and often undetected) interaction, using this model leads possibly to a biased and/or less powerful analysis [26].

Furthermore, with this model, estimation of the exposure effect cannot be adjusted for a possible interaction between the matching factors and the exposure. This stratified approach is sensitive to the unit number per strata and to the number of strata: the accuracy of the regression coefficients decreases for a small number of units per strata and/or many numbers of strata [27].

This model is implemented in R software [28] through the *coxph* function by including the term “*strata(P*_*i*_(*t*))” with the other explanatory covariates.

#### Lee, Wei and Amato Cox model

The marginal *LWA* model [20] is an alternative to the standard Cox model [11] and is written as follows

$$\lambda_{i}\left(t,Z_{i}\right)=\lambda_{0}\left(t\right)\exp\left(\gamma \left(t\right)E_{i}\left(t\right)\right),\;({\text{LWA}}_{u})$$

if the exposure effect is not adjusted for the matching covariates vector **Z**;

$$\lambda_{i}\left(t,Z_{i}\right)=\lambda_{0}\left(t\right)\exp\left(\beta^{\prime }Z_{i}+\gamma \left(t\right)E_{i}\left(t\right)\right),\;({\text{LWA}}_{a})$$

if the exposure effect is adjusted for the matching covariates vector **Z**;

$$\begin{array}{ll} \lambda_{i}\left(t,Z_{i}\right) & =\lambda_{0}\left(t\right)\exp\left(\beta^{\prime }Z_{i}+\gamma \left(t\right)E_{i}\left(t\right)+\alpha^{\prime }\left(t\right)Z_{i}E_{i}\left(t\right)\right),\\ & \left({\text{LWA}}_{i}\right) \end{array}$$

if the exposure effect is adjusted for the matching covariates vector **Z**, and for the interaction between **Z** and the exposure.

For each of these three *LWA* models, $\lambda_{0}\left(t\right)$ is an unspecified marginal baseline hazard function considered as common for all the pairs, so for the whole population. As above, it is considered as a nuisance parameter; $\exp\left(\gamma \left(t\right)\right)$ is the average time-varying exposure effect as in the *HP* model, but adjusted (*LWA*_*a*_) or not (*LWA*_*u*_) for covariates **Z** and for the possible interaction between covariates and exposure (*LWA*_*i*_). Like the standard Cox model [11], the *LWA* assumes that all sample subjects are homogeneous (all subjects have the same $\lambda_{0}\left(t\right)$) in spite of the possible adjustment for covariates (unique difference between *LWA*_*u*_ and *HP*).

This model is implemented in R software through the *coxph* function, by including the term “*cluster(P*_*i*_(*t*))” with the other explanatory covariates.

For both models, the Proportional Hazard Assumption (PHA) was evaluated by Harrel’s test on scaled Schoënfeld residues. This test is implemented in R software through the *cox.zph* function. The possible time-dependent effect of the exposure was taken into account by time intervals chosen *a posteriori*, and not by a time-specified function.

Note that the combination *HP* and Method 1, taking the exposure as a time-dependent covariate or not, gave exactly the same estimation of $\bar{\text{HR}}\left(t\right)$, whereas *LWA* did not.

Table 1 presents all the models according to the adjustment or not for covariates.

**Table 1:** $\lambda_{i}(t,Z_{i})$** estimations with the *HP * and *LWA * models are associated to the *HR(t) * to be estimated according to the adjustment for covariates**

**Exposure covariate**	**Models**		***H R *** **( *t *)**
	***H*** ***P***	**LWA**	
	***λ***_***i***_**( *t *,Z**_***i***_**)**	***λ***_***i***_**( *t *,Z**_***i***_**)**	
Time-dependent			
and no time-dependent effect	$\lambda_{0i(j)}(t)\exp\left(\gamma E_{i}\left(t\right)\right)$	$\lambda_{0}(t)\exp\left(\gamma E_{i}\left(t\right)\right)$	$\bar{\text{HR}}(t)$
With adjustment for **Z**		*λ*_0_(*t*)exp(*γE*_*i*_(*t*)+ ***β***^′^**Z**_*i*_)	${\bar{\text{HR}}}_{a}(t)$
and with interaction		*λ*_0_(*t*)exp(*γE*_*i*_(*t*)+ ***β***^′^**Z**_*i*_+ ***α***^′^(*t*)**Z**_*i*_*E*_*i*_(*t*))	*HR*_*i*_(*t*)
Time-dependent			
and time-dependent effect	$\lambda_{0i(j)}(t)\exp\left(\gamma \left(t\right)E_{i}\left(t\right)\right)$	$\lambda_{0}(t)\exp\left(\gamma \left(t\right)E_{i}\left(t\right)\right)$	$\bar{\text{HR}}(t)$
With adjustment for **Z**		*λ*_0_(*t*)exp(*γ*(*t*)*E*_*i*_(*t*)+ ***β***^′^**Z**_*i*_)	${\bar{\text{HR}}}_{a}(t)$
and with interaction		*λ*_0_(*t*)exp(*γ*(*t*)*E*_*i*_(*t*)+ ***β***^′^**Z**_*i*_+ ***α***^′^(*t*)**Z**_*i*_*E*_*i*_(*t*))	*HR*_*i*_(*t*)

*λ*_*i*_(*t*,**Z**_*i*_) is the instantaneous risk function of event for the pair *P*_*j*_, which is composed of the exposed subject *j* and the non-exposed subject *i* matching on **Z**_*i*_.

In the following, different simulations and analyses were performed with R software version 2.13.0.

## Results

### Simulations

#### Objective

The main objective of the simulation study was to assess the ability of the *HP* and *LWA* models to estimate the true effect of exposure *HR*(*t*), defined by $\exp\left(\gamma \left(t\right)\right)$, in a context of matched paired survival data, where the pairs were designed according to the two different methods described previously. The aim was to establish the most efficient Method - Model combination.

#### Dataset

##### Simulation of cohort data - Procedures and scenarios chosen

All the details of the cohort data simulation and the procedures and scenarios chosen are given in Appendix A.

**Figure 1:**
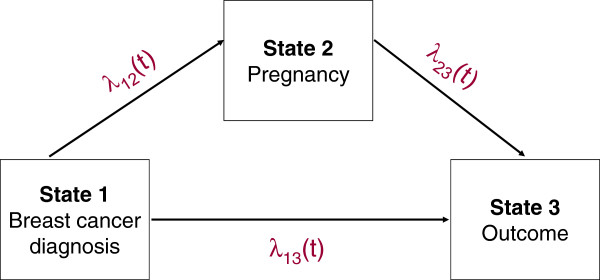
**“Illness-death” model.** “Illness-death” model with three transition intensities $\lambda_{\text{uv}}(t)$.

We simulated the cohort data referring to an “illness-death” model with transition intensities $\lambda_{12}\left(t\right)$, $\lambda_{13}\left(t\right)$ and $\lambda_{23}\left(t\right)$ (Figure 1). The parameter of interest *HR*(*t*) corresponded to the ratio $\lambda_{23}\left(t\right)/\lambda_{13}\left(t\right)$. The average *HR*(*t*) is obtained from an exact formula involving the averages of $\lambda_{13}\left(t\right)$ and $\lambda_{23}\left(t\right)$ which are computed through a numerical approximation (transformation of the time from continuous to discrete values) (See the Appendix B: Calcul of the average exposure effect not adjusted for the covariates). The average *HR*(*t*) adjusted for the different covariates was estimated empirically: its estimation was obtained using large size samples to guarantee good precision. Moreover, note that the larger the ratio $\lambda_{12}\left(t\right)/\lambda_{13}\left(t\right)$, the larger the number of exposures in the simulated cohort.

Each subject was characterized by a prognostic profile through vector **Z**, which corresponded to three dummy covariates *Z*_*k*_ (*k*=1,2 or 3). At time *t*=0, there were 2^3^=8 possible profiles of **Z** factors.

The simulation model included (i) the choice of an instantaneous baseline risk function $\lambda_{\text{uv}}\left(t,Z\right)$ for each of the three transitions u→v (Table 2), (ii) the choice of the **Z** effects, $\exp\left(\beta_{\text{uvk}}\right)$, for each transition, *i.e.*$\lambda_{\text{uv}}\left(t,Z\right)=\lambda_{0,\text{uv}}\left(t\right)\exp\left(\beta_{\text{uv}1}Z_{1}+\beta_{\text{uv}2}Z_{2}+\beta_{\text{uv}3}Z_{3}\right)$ and (iii) the choice for the censoring proportion.

**Table 2:** Survival functions applied to simulate each transition and each selected configuration

	**Constant**$\text{HR}{\left(t\right)}^{*}$	**Increasing** $$\text{HR}\left(t\right)$$	**Decreasing** $$\text{HR}\left(t\right)$$	**Increasing then**
				**decreasing** $\text{HR}\left(t\right)$
Transition 1→2	Exponential ^(1)^	Exponential ^(1)^	Exponential ^(1)^	Exponential ^(1)^
	$\left(\lambda \right)$	$\left(\lambda \right)$	$\left(\lambda \right)$	$\left(\lambda \right)$
	$\left(0.0050\right)$	$\left(0.0075\right)$	$\left(0.0025\right)$	$\left(0.0020\right)$
Transition 1→3	Weibull ^(2)^	Weibull ^(2)^	Loglogistic ^(3)^	Weibull ^(2)^
	$\left(\lambda_{0},\gamma \right)$	$\left(\lambda_{0},\gamma \right)$	$\left(\mu ,\sigma \right)$	$\left(\lambda_{0},\gamma \right)$
	$\left(0.0039,1.1881\right)$	$\left(0.0022,1.1881\right)$	$\left(5.7146,0.2390\right)$	$\left(0.0018,1.1881\right)$
Transition 2→3	Weibull ^(2)^	Weibull ^(2)^	Loglogistic ^(3)^	Loglogistic ^(3)^
	$\left(\lambda_{0},\gamma \right)$	$\left(\lambda_{0},\gamma \right)$	$\left(\mu ,\sigma \right)$	$\left(\mu ,\sigma \right)$
	$\left(0.0039,1.1881\right)$	$\left(0.0028,1.5439\right)$	$\left(5.6778,0.2463\right)$	$\left(5.9858,0.4971\right)$

^(1)^$\bar{S}(t)=\exp(-\mathrm{\lambda t});{\;}^{\left(2\right)}\bar{S}(t)=\exp(-{\left(\lambda_{0}t\right)}^{\gamma });{\;}^{\left(3\right)}\bar{S}(t)={\left[1+\exp(\frac{\ln(t)-\mu }{\sigma })\right]}^{-1}$.

^*^We simulated the same functions and parameters for the second Constant *HR*(*t*) except for Transition 2→3 where *λ*_0_=0.0069.

For (i), an instantaneous average risk function $\lambda_{\text{uv}}\left(t,Z=\bar{Z}\right)$ for each of the three transitions was simulated. Table 2 displays the $\lambda_{\text{uv}}\left(t,Z=\bar{Z}\right)$ distributions of each transition used for each of the five different configurations of *HR*(*t*).

For (ii), ten different *β*_*u* *v* *k*_ scenarios considered as plausible *β*_*u* *v* *k*_ clinical values [9,15], were performed. Given the five configurations chosen for *HR*(*t*) and these ten *β*_*u* *v* *k*_ scenarios, 50 different situations were obtained.

Finally, for (iii), these previous 50 situations were first performed without censoring. Two levels of independent uniform censoring were implemented only to the following *β*_*u* *v* *k*_ scenario: ${\;\beta }_{12}^{{}^{\prime }}=\left(-0.2,-0.4,-0.8\right)$, ***β***_13_=−***β***_12_ and ***β***_23_= ***β***_12_; and they were applied to each of the five configurations of *HR*(*t*). This yielded to 10 more situations.

For each of the 60 situations, 1000 different data sets were generated with a sample size of 2000 subjects. At *t*=0, these 2000 subjects were allocated to eight **Z** profiles. At *t*>0, the 250 subjects of the 8 different profiles will be divided up in the three transitions and will change over time according to the five *HR*(*t*) configurations.

All theoretical values of *HR*(*t*) were calculated on the simulated cohort data. They were computed in the overall correlated censored data and inside each sample of the **Z** profile. The average *HR*(*t*) was calculated without and with adjustment for the matching covariates, *i.e.*$\bar{\text{HR}}\left(t\right)$ and ${\bar{\text{HR}}}_{a}\left(t\right)$ respectively. *HR*_*i*_(*t*) is the one estimated in each **Z** profile.

From the correlated censored data, *HP* and *LWA*_*u*_ models are both assumed to give an average *HR*(*t*)*i.e.*$\bar{\text{HR}}\left(t\right)$ (considering different assumptions), so they are the only two models which could be compared. Fitting of model *LWA*_*a*_ makes it possible to estimate an average *HR*(*t*)*i.e.*${\bar{\text{HR}}}_{a}\left(t\right)$, while *LWA*_*i*_ is assumed to give *HR*_*i*_(*t*) for each **Z** profile (Table 1).

As the exposure effect was considered to change over time for three of the five configurations, its estimation was assessed by time interval specified *a posteriori*.

##### Matching methods - Creation of censored correlated data from cohort data

For each data set, the two matching methods presented in Section ‘Methods’ were applied.

According to Methods 1 and 2, the two subjects in each pair were matched on the three covariates *Z*_*k*_, and the non-exposed subject had to be disease-free for as long as the time from *t*=0 to exposure time of the exposed subject.

Then from the 2000 subjects simulated in cohort data sets and equally allocated to the 8 **Z** profiles, a number of pairs smaller or equal to the number of subjects in State 2 (*i.e.* pregnancy) were obtained. This latter depended on the situation simulated, resulting from the *HR*(*t*) configuration, the *β*_*u* *v* *k*_ scenario and the censoring percent.

##### Statistical criteria used to compare the performances of the different estimators

To estimate a time-dependent effect, the time interval $\left[\;0-t_{\max}\right]$ was divided into *L* time intervals *I*_*l*_ defined *a priori*, according to the *HR*(*t*) configuration, and written as follows:

$$a_{0}=0<a_{1}<\ldots <a_{L}=t_{\max}$$

and

$$I_{l}=\;[\;a_{l-1};a_{l}[\;,l\geq 1.$$

If there was no interaction and no time-dependent effect, an estimation ${\hat{\gamma }}_{s}$ was obtained that corresponded to the estimation of *γ* performed in the *s*^*t* *h*^ simulation set inside the same *HR*(*t*) configuration.

If there was an interaction and no time-dependent effect, an estimation for each of the 8 **Z** prognostic profiles was obtained and expressed as ${\hat{\gamma }}_{s}+{\hat{\alpha }}_{s}^{{}^{\prime }}Z$.

If there was no interaction and a time-dependent effect, an estimation for each of the *I*_*l*_ time intervals was obtained and expressed as ${\hat{\gamma }}_{\text{sl}}$.

If there was an interaction and a time-dependent effect, 8×*L* estimations were obtained and expressed as ${\hat{\gamma }}_{\text{sl}}+{\;\hat{\alpha }}_{\text{sl}}^{{}^{\prime }}Z$.

To assess the combination Method - Model to estimate *HR*(*t*), the bias and the *RMSE* of the estimations presented above were calculated. The 8 **Z** profiles are indexed by *w*=1 to 8, and the profile w is noted **Z**^(*w*)^.

In the event of with an interaction and a time-dependent effect, the bias was estimated for each *l* and each *Z*^(*w*)^ over the 1000 simulations as follows:

$$\begin{array}{ll} b_{lZ^{\left(w\right)}} & =\frac{1}{1000}\overset{1000}{\underset{s=1}{\sum }}\left({\hat{\gamma }}_{\text{sl}}+{\hat{\alpha }}_{\text{sl}}^{{}^{\prime }}Z-\left(\gamma_{l}+\alpha_{l}^{{}^{\prime }}Z\right)\right)\\ & =\bar{\gamma_{l}+\alpha_{l}^{{}^{\prime }}Z}-\left(\gamma_{l}+\alpha_{l}^{{}^{\prime }}Z\right) \end{array}$$

and

$${\text{RMSE}}_{lZ^{\left(w\right)}}=\sqrt{b_{lZ^{\left(w\right)}}^{2}+V\left({\hat{\gamma }}_{l}+{\hat{\alpha }}_{l}^{{}^{\prime }}Z\right)},$$

where, in each *I*_*l*_ interval, $\bar{\gamma_{l}+\alpha_{l}^{{}^{\prime }}Z}$ is the mean and $V\left({\hat{\gamma }}_{l}+{\hat{\alpha }}_{l}^{{}^{\prime }}Z\right)$ the empirical variance of the 1000 parameters estimated ${\hat{\gamma }}_{\text{sl}}+{\hat{\alpha }}_{\text{sl}}^{{}^{\prime }}Z$:

$$\begin{array}{ll} V\left({\hat{\gamma }}_{l}+{\hat{\alpha }}_{l}^{{}^{\prime }}Z\right)= & \;\frac{1}{\left(1000-1\right)}\left[\sum_{s=1}^{1000}{\left({\hat{\gamma }}_{\text{sl}}+{\hat{\alpha }}_{\text{sl}}^{{}^{\prime }}Z\right)}^{2}\right)\\ & \left(-\;\frac{{\left[\overset{1000}{\underset{s=1}{\sum }}\left({\hat{\gamma }}_{\text{sl}}+{\hat{\alpha }}_{\text{sl}}^{{}^{\prime }}Z\right)\right]}^{2}}{1000}\right]. \end{array}$$

To compare the different combinations Method - Model, the bias was averaged over the profiles (*b*_*l*•_) or over the time intervals $\left({b_{•Z}}^{\left(w\right)}\right)$ or both (*b*_••_).

$$\begin{array}{lcrr} b_{•Z^{\left(w\right)}} & = & \frac{1}{L}\overset{L}{\underset{l=1}{\sum }}b_{l{\;Z}^{\left(w\right)}} & \\ b_{l•} & = & \frac{1}{8}\overset{8}{\underset{w=1}{\sum }}b_{lZ^{\left(w\right)}} & \\ b_{••} & = & \frac{1}{8}\overset{8}{\underset{w=1}{\sum }}b_{•Z^{\left(w\right)}} & \end{array}$$

The associated *RMSE*s are given by

$$\begin{array}{lcrr} {\text{RMSE}}_{•Z^{\left(w\right)}} & = & \sqrt{\frac{1}{L}\overset{L}{\underset{l=1}{\sum }}b_{lZ^{\left(w\right)}}^{2}+V\left({\hat{\gamma }}_{l}+{\hat{\alpha }}_{l}^{{}^{\prime }}Z\right)} & \\ {\text{RMSE}}_{l•} & = & \sqrt{\frac{1}{8}\overset{8}{\underset{w=1}{\sum }}b_{lZ^{\left(w\right)}}^{2}+V\left({\hat{\gamma }}_{l}+{\hat{\alpha }}_{l}^{{}^{\prime }}Z\right)} & \\ {\text{RMSE}}_{••} & = & \sqrt{\frac{1}{8}\overset{8}{\underset{w=1}{\sum }}\frac{1}{L}\overset{L}{\underset{l=1}{\sum }}b_{lZ^{\left(w\right)}}^{2}+V\left({\hat{\gamma }}_{l}+{\hat{\alpha }}_{l}^{{}^{\prime }}Z\right)} & \end{array}$$

Note that if the exposure effect is not time-dependent, then *L*=1. If there is no interaction between the exposure and the prognostic profiles, then *α*=0.

#### Results

**Figure 2:**
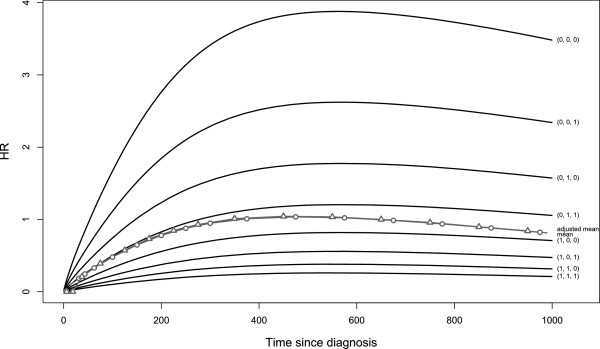
***HR(t)*configuration chosen.** Increasing then decreasing *HR*(*t*) configuration, for each **Z** profile and on average, without censoring and with $\beta_{12}^{{}^{\prime }}=\left(-0.2,-0.4,-0.8\right)$, $\beta_{13}=-\beta_{12}$ and $\beta_{23}=-\beta_{13}$. This figure displays the theoretical estimations of $\bar{\text{HR}}\left(t\right)$ called “mean”, ${\bar{\text{HR}}}_{a}(t)$ called “adjusted mean” and *HR*_*i*_(*t*) in the eight prognostic profiles. The profile $Z^{{}^{\prime }}=(0,0,0)$ at time *t*=0 is the profile with the better prognosis; the profile $Z^{{}^{\prime }}=(1,1,1)$ has the worse prognosis, and the 6 others an intermediate prognosis. In this particular configuration chosen, where $\beta_{23}=-\beta_{13}$, $\bar{\text{HR}}\left(t\right)≃{\bar{\text{HR}}}_{a}\left(t\right)$ and their values are so close that the difference between them is not visible in this figure.

One particular situation among the 60 simulated is described. Figure 2 displays the increasing then decreasing *HR*(*t*) configuration, for each profile and on average, without censoring and with $\beta_{12}^{{}^{\prime }}=\left(-0.2,-0.4,-0.8\right)$, ***β***_13_=−***β***_12_ and ***β***_23_=−***β***_13_. In this particular situation which corresponds to a “healthy effect” because of the negative values of *β*_12_, Figure 2 shows three different overall effects of the exposure: a pejorative one in the three better prognostic profiles (PP) ($Z^{{}^{\prime }}\in \left\{\left(0,0,0\right),\left(0,0,1\right),\left(0,1,0\right)\right\}$), no effect in the intermediate PP ($Z^{{}^{\prime }}\in \left\{\left(0,1,1\right)\right\}$) and a protective effect in the last four PP ($Z^{{}^{\prime }}\in \left\{\left(1,0,0\right),\left(1,0,1\right),\left(1,1,0\right),\left(1,1,1\right)\right\}$). With ***β***_23_=−***β***_13_, we force an interaction between **Z** and the exposure. Note that in this particular configuration chosen, where ***β***_23_=−***β***_13_, $\bar{\text{HR}}\left(t\right)≃{\bar{\text{HR}}}_{a}\left(t\right)$ and their values are so close that the difference between them is not visible in Figure 2.

##### Number of pairs

**Figure 3:**
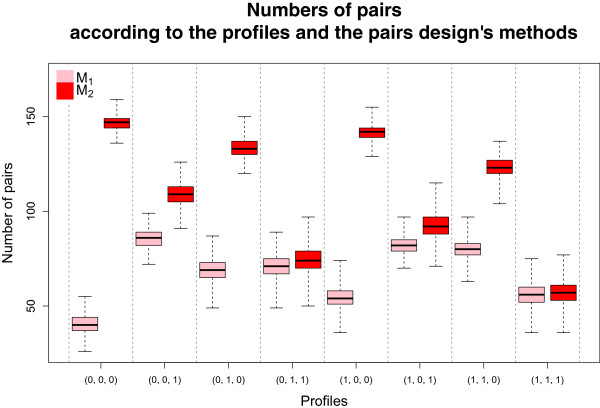
**Number of pairs.** Distribution of the number of pairs according to the profiles and to the matching methods *M*_1_ and *M*_2_. Results obtained with the increasing then decreasing *HR*(*t*) configuration, without censoring and with $\beta_{12}^{{}^{\prime }}=(-0.2,-0.4,-0.8)$, ***β***_13_=−***β***_12_ and $\beta_{23}=-\beta_{13}$.

Inside each profile, the maximum number of pairs was determined by the number of exposed subjects. With Method 1, this number was also limited by the number of “perfect” non-exposed subjects, but not with Method 2 since the non-exposed subject set was composed of “perfect” and “imperfect” non-exposed subjects. The difference between the number of pairs from the two methods depended on the number of exposures: the larger the number, the larger the difference. We computed the relative difference (*RD*) in number of pairs between the two methods defined as

$$\begin{array}{c} \;\text{RD}=\frac{\text{Number of pairs with Method}2-\text{Number of pairs with Method}1}{\text{Number of pairs with Method}1}, \end{array}$$

whose median was equal to +55*%* (range, +23*%* to +220*%*). Figure 3 represents the distribution of the number of pairs according to the profiles and to the matching methods: the median number with Method 2 was always larger than or equal to that with Method 1.

**Figure 4:**
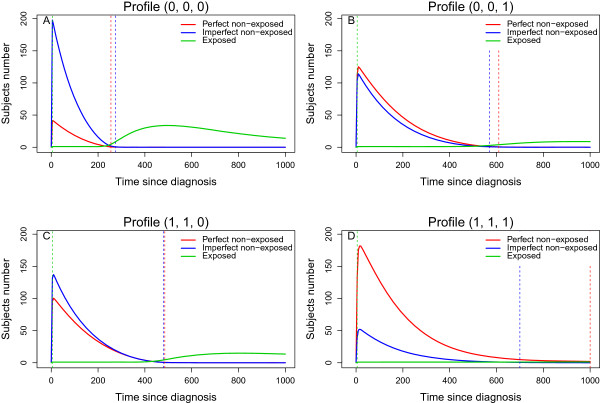
**Number of subjects in the three possible groups.** Number of subjects pertaining to the three possible subjects groups at each time *t*: the exposed subject (solid green line), the non-exposed subject who never will undergo the exposure (solid red line) and the non-exposed subject who will undergo the exposure (solid blue line). The dotted vertical green line represents the time of first exposure, *i.e.* the time of occurrence of the first pair; the dotted vertical blue line corresponds the time of the last perfect pair’s creation; and the dotted vertical red line corresponds the time of the last imperfect pair’s creation. Results obtained with the increasing then decreasing *HR*(*t*) configuration, without censoring and with $\beta_{12}^{{}^{\prime }}=(-0.2,-0.4,-0.8)$, $\beta_{13}=-\beta_{12}$ and $\beta_{23}=-\beta_{13}$.

Figures 4 shows the number of subjects pertaining to the three possible subjects groups at each time *t*: the exposed subject (solid green line), the non-exposed subject who never will undergo the exposure (solid red line) and the non-exposed subject who will undergo the exposure (solid blue line). The dotted vertical green line represents the time of first exposure, *i.e.* the time of occurrence of the first pair; the dotted vertical blue line corresponds to the time of the last perfect pair’s creation and the dotted vertical red line corresponds to the time of the last imperfect pair’s creation. With Method 2, the larger the ratio $\lambda_{12}\left(t\right)/\lambda_{13}\left(t\right)$, the larger the number of imperfect pairs and thus the greater the probability for an exposed subject to belong to an imperfect pair. Table 3 provides the proportion of imperfect pairs among the whole pairs which was estimated over the 1000 simulated data sets of our particular situation. It was equal to 81*%* in the good profile $Z^{{}^{\prime }}=(0,0,0)$, decreasing to 54*%*, 44*%* and 20*%* in the **Z** profiles (1,1,0), (0,0,1) and (1,1,1), respectively. Moreover, the larger the ratio $\lambda_{12}\left(t\right)/\lambda_{13}\left(t\right)$, the faster the pair’s creation stopped. After the last dotted line (blue or red, depending on the **Z** profile), the exposed subjects are no longer able to be matched with a non-exposed one, because they are no longer available subjects; the pair’s creation stopped at a time that gradually increased from $Z^{{}^{\prime }}=(0,0,0)$ to $Z^{{}^{\prime }}=(1,1,1)$. For instance, this is illustrated for profile $Z^{{}^{\prime }}=(0,0,0)$ in Figure 4A: at the time of first exposure (dotted vertical green line), the exposed subject was more likely to be matched with an imperfect subject than to a perfect non-exposed one. This set of non-exposed subjects decreased over time because each of them was matched with an exposed subject until the dotted blue line, when no more non-exposed subjects were available, while a new exposed subject, belonging before to a pair as a non-exposed one, appeared. This set of exposed subjects increased and was not able to be matched because there were no longer any non-exposed subjects. For a same level of $\lambda_{12}\left(t\right)/\lambda_{13}\left(t\right)$ ratio, RD was larger depending on the censoring percent: the higher the censoring percent, the smaller the RD.

**Table 3:** **Simulations results: time-dependent bias**$b_{•Z^{\left(w\right)}}$** with**${\text{RMSE}}_{•Z^{\left(w\right)}}$** for the** ***LWA***_***i***_** model and the mean of the number of pairs inside each prognostic profile and according to the matching methods are presented with the percent of imperfect pairs for Method 2**

**Prognostic profiles Z**^**( *w *)**^	**% of exposure**	**Method 1**				**Method 2**		
		$b_{•Z^{\left(w\right)}}$	${\text{RMSE}}_{•Z^{\left(w\right)}}$	**Mean of pairs number**	$b_{•Z^{\left(w\right)}}$	${\text{RMSE}}_{•Z^{\left(w\right)}}$	**Mean of pairs number**	**% of imperfect pairs**
(0,0,0)	0.82	-0.97	1.05	40.26	0.13	0.32	146.88	0.81
(0,0,1)	0.48	-0.46	0.59	85.46	0.04	0.33	109.05	0.44
(0,1,0)	0.67	-0.70	0.79	68.82	0.08	0.31	133.47	0.64
(0,1,1)	0.30	-0.22	0.46	70.87	-0.03	0.36	74.07	0.26
(1,0,0)	0.76	-0.83	0.91	54.48	0.10	0.31	141.70	0.73
(1,0,1)	0.39	-0.34	0.51	82.27	0.007	0.34	92.61	0.35
(1,1,0)	0.58	-0.57	0.67	79.94	0.05	0.31	123.44	0.54
(1,1,1)	0.23	-0.09	0.46	56.15	-0.06	0.39	56.88	0.20

The percent of exposure inside each prognostic profile is also given.

##### Bias and ***RMSE***

**Figure 5:**
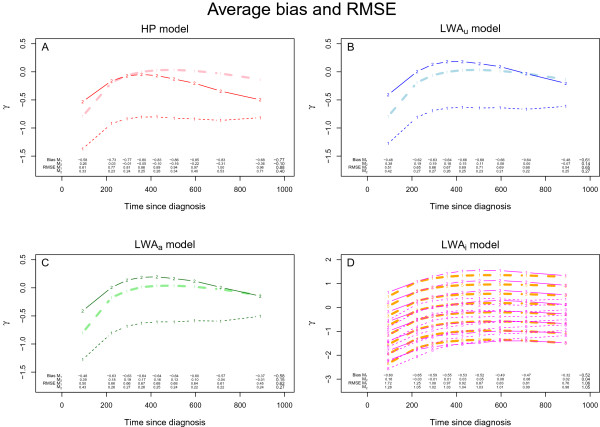
**Methods and Models.** Exposure effect’s estimation according to Methods 1 and 2 and related to: **(A)** The *HP* model, **(B)** The *LWA*_*u*_ model, **(C)** The *LWA*_*a*_ model and **(D)** The *LWA*_*i*_ model. The solid thick lines red, blue and green (Figure A, B and C) respectively, represent the theoretical average of γ(t) and the solid thick orange lines (Figure D) represent the theoretical $\gamma (t)+\alpha^{{}^{\prime }}(t)Z$ inside each profile **Z**. The fine lines red, blue and green (Figure A, B and C) respectively, represent γ(t), estimated according to Methods 1 (fine dotted lines) and 2 (solid dotted lines); and the fine red lines (Figure D) represent the observed $\gamma (t)+\alpha^{{}^{\prime }}(t)Z$ inside each profile. The average bias and *RMSE* are given for each model: *b*_*l*•_, *b*_••_, *RMSE*_*l*•_ and *RMSE*_••_. These results are obtained with the increasing then decreasing *HR*(*t*) configuration, without censoring and with $\beta_{12}^{{}^{\prime }}=(-0.2,-0.4,-0.8)$, $\beta_{13}=-\beta_{12}$ and $\beta_{23}=-\beta_{13}$.

Figures 5 displays the distribution of the bias and the *RMSE* of the exposure effect estimator, according to our four models.

With Method 1, the estimation of $\bar{\text{HR}}\left(t\right)$ was biased with *HP* and *LWA*_*u*_, but more with *HP*; the estimation of ${\bar{\text{HR}}}_{a}\left(t\right)$ was biased with *LWA*_*a*_. Whatever the model applied, $\bar{\text{HR}}\left(t\right)$ (*HP*, *LWA*_*u*_) and ${\bar{\text{HR}}}_{a}\left(t\right)({\text{LWA}}_{a})$ were underestimated, *i.e.* a poor prognostic exposure effect tended to be ignored and a no exposure effect tended to become a protective one. In each **Z** profile, *LWA*_*i*_ always largely underestimated *HR*_*i*_(*t*) (Figures 5). The bias *b*_••_ is equal to −0.77, −0.61, −0.58 and −0.52 under *HP*, *LWA*_*u*_, *LWA*_*a*_ and *LWA*_*i*_ models, respectively; and the *RMSE*_••_ is equal to 0.88, 0.65, 0.62 and 1.06 under *HP*, *LWA*_*u*_, *LWA*_*a*_ and *LWA*_*i*_ models, respectively (Figures 5). The bias and *RMSE* are given by PP in Table 3.

With Method 2 where $\bar{\text{HR}}\left(t\right)≃{\bar{\text{HR}}}_{a}\left(t\right)$, *LWA*_*u*_ and *LWA*_*a*_ gave very similar values, whereas *HP* gave a smaller estimation of $\bar{\text{HR}}\left(t\right)$ than *LWA*_*u*_. All these three models are very close to the true value of the effect of exposure, but none gave an unbiased estimation: this estimation was slightly over- or under-estimated according to the time, but much less than with Method 1. *HR*_*i*_(*t*) was overestimated in some **Z** profiles: *HR*_*i*_(*t*) was overestimated in the three PP, $Z^{{}^{\prime }}\in \left\{\left(0,0,0\right),\left(1,0,0\right),\left(0,1,0\right)\right\}$, where the ratio *λ*_12_(*t*)/*λ*_13_(*t*) was large and then the proportion of imperfect pairs in the set of pairs to be analyzed, was also large (Figures 5). The mean of the time-dependent bias *b*_••_ was equal to -0.10, 0.14, 0.15 and 0.04 under *HP*, *LWA*_*u*_, *LWA*_*a*_ and *LWA*_*i*_ models, respectively; and *RMSE*_••_ was equal to 0.40, 0.27, 0.27 and 1.05 under *HP*, *LWA*_*u*_, *LWA*_*a*_ and *LWA*_*i*_ models, respectively (Figures 5). The biases and *RMSE* are quite acceptable and much smaller than with Method 1. Table 3 displays the bias and *RMSE* more specifically, with the percent of imperfect pairs according to the PP: the larger the percentage of imperfect pairs, the larger the bias and *RMSE*.

All the conclusions displayed with Method 1 were the same for all five configurations and all the *β*_*u* *v* *k*_ triplet values. The biases of $\bar{\text{HR}}\left(t\right)$, ${\bar{\text{HR}}}_{a}\left(t\right)$ and ${\text{HR}}_{i}\left(t\right)$ were always huge (data not shown) but more or less followed the configuration and the *β*_*u* *v* *k*_ scenarios. All the conclusions with Method 2 were valid for all configurations, and for all *β*_*u* *v* *k*_ triplet values. In the configurations without interaction, *i.e.* where ***β***_23_= ***β***_13_, *HP* and *LWA*_*u*_ models were more appropriate than *LWA*_*a*_ and *LWA*_*i*_ to estimate $\bar{\text{HR}}\left(t\right)$ in terms of bias and *RMSE*, given that *LWA*_*u*_ was much less biased than *HP* in most of the scenarios. In some configurations where the proportion of the profile with the smallest ${\bar{\text{HR}}}_{i}\left(t\right)$ was the most highly represented profile leading to a low $\bar{\text{HR}}\left(t\right)$, *HP* was better than *LWA*_*u*_. *LWA*_*a*_ was the only model for estimating *HR*_*a*_(*t*) and led to very slightly biased estimations of ${\text{HR}}_{a}\left(t\right)$ (data not shown). In the configurations with interaction, *i.e.* where ***β***_23_≠ ***β***_13_, the *LWA*_*i*_ model was the only appropriate one. However, in some of these configurations, *LWA*_*i*_ slightly biased the extreme profiles and not the intermediate ones (data not shown). Over the 10 situations with censoring, the censoring percent ranged from 9% to 48%. Censoring did not change any of the previous conclusions.

Overall, in terms of bias and *RMSE*, Method 2 performed better than Method 1 to design the pairs, and *LWA*_*a*_ was the best model for all the situations except when there was an interaction between the covariates and the exposure (***β***_23_≠ ***β***_13_), for which *LWA*_*i*_ was more appropriate, even if the estimations with *HR*_*i*_(*t*) were not uniformly unbiased.

### Real data application

#### Data

Our data retrospectively included 870 women treated for breast cancer between January 1990 and December 1999, and diagnosed before 35 years of age. Information on patients’ status was collected at the end of the year 2004 and the median follow-up was 87 months (range, 7 to 166) at this date. Tumor, treatment and disease evolution analyses are available in the paper of Largillier *et al.*[9]. One of the goals of the data analysis was to compare disease-free survival between pregnant and non-pregnant patients. The protocol was submitted to the appropriate French authorities supervising individual computerized data files (Commission Nationale Informatique et Liberté [CNIL]), and obtained ethical approval from the Institut Curie ethics committee (Comité des Etudes en Recherche Clinique [CERC]). The GETNA Working Group, which was responsible for the data conception, design and acquisition, allowed us access to these real data.

We created pairs of pregnant and non-pregnant women using the two matching methods. Both women were matched according to their cancer gravity level using: Scarff-Bloom-Richardson grade (*SBR*, related to cell proliferation level), pathological node involvement and hormonal receptor status (*RH*). They were also matched on treatment: hormonotherapy prescribed or not to RH+ patients, and chemotherapy administered or not before and/or after surgery. Within each pair, the non-pregnant woman had to be disease-free for as long as the time from diagnosis to pregnancy of the pregnant woman. We sought to estimate the effect of subsequent pregnancy occurring over time on breast cancer evolution.

According to the results obtained on the cohort data [15] and to the known breast cancer clinical prognostic factors in the literature, patients with low cell proliferation level (*SBR* I or II) and no node involvement were considered to have a good prognostic profile and likely to plan to be pregnant (“healthy mother effect”). According to biological assumptions and need for therapy (hormonal treatment if *RH*+), women with negative hormonal receptors were also more likely to be pregnant. Thus, even if these factors were not significant in the paper by Savignoni *et al.*[15], it seemed relevant to estimate the effect of pregnancy according to the *RH* status (and the treatment associated) and according to a clinical Prognostic Profile (*cPP*); a poor *cPP* was defined as an *SBR* grade III and/or pathological involved nodes, leading to six prognostic profiles: *RH* negative with a good *cPP*, *RH* negative with a poor *cPP*, *RH* positive not treated with a good *cPP*, *RH* positive not treated with a poor *cPP*, *RH* positive treated with a good *cPP* and *RH* positive treated with a poor *cPP*. Then the effect of the pregnancy was estimated in the whole population by adjusting or not for the matching factors and with regard to the six prognostic profiles by adjusting for an interaction between the pregnancy, and the *RH* status (associated with treatment) and the *cPP* respectively. The effect of pregnancy was not adjusted for chemotherapy treatment.

#### Results

In view of our simulations, Method 2 was the matching method to apply on the cohort data in order to create correlated censored data that would give a more accurate estimate of the exposure effect. To be able to compare our results with previous findings [3]-[5,7,10], we used the *HP* model on correlated censored data designed from Method 1.

First, we applied the Method 1—*HP* combination as proposed in the literature [3]-[5,7,10]. Secondly, we applied the *HP* and *LWA* with pregnancy and pair as time-dependent covariates on the paired survival data created with Method 2. With *LWA*, $\bar{\text{HR}}\left(t\right)$ estimation was carried out (i) without adjusting for matching covariates (*LWA*_*u*_ estimates $\bar{\text{HR}}\left(t\right)$), (ii) by adjusting for all the matching covariates but without any interaction (*LWA*_*a*_ estimates ${\bar{\text{HR}}}_{a}\left(t\right)$) and (iii) by adjusting for all the matching covariates and with an interaction between pregnancy and matching covariates (*LWA*_*i*_ estimates *HR*_*i*_(*t*)). The latter could be applied in the event of real or assumed biological and clinical interactions. We tested the PHA with Harrel’s test on the two correlated censored data sets and with each model. The PHA was verified and we did not add any time-effect for pregnancy in the models. *HR*(*t*) was compared to 1 by the Wald’s test with the appropriate variance according to the models.

In the cohort data, only 668 patients presented no missing data for the covariates of interest. Among them, 68 experienced a subsequent pregnancy. We obtained the maximum number of pairs available with Methods 1 and 2, *i.e.* 68 pairs: all pregnant women were then matched. The 68 pairs were not exactly the same between Methods 1 and 2. In Method 2 five pairs were imperfect, which represented a low proportion (7.4*%*). Among the 68 pairs obtained with Method 1, 32 women experienced an event (progression or death): 16 in the group of patients who became pregnant after the breast cancer diagnosis and 16 in the group of patients who did not. Among the 68 pairs obtained with Method 2, 29 women experienced an event: the same 16 in the group of patients who became pregnant after the breast cancer diagnosis and 13 in the group of patients who did not. No events occurred in the imperfect pairs. Only sixteen pairs (23.5*%*) were common between the two matching methods. The number of pairs and the number of final events in the pregnancy and non-pregnancy groups are given Table 4, according to the six profiles and to the matching methods. The number of pairs was divided into imperfect and perfect pairs for Method 2.

**Table 4:** Real data: number of pairs and final events according to the matching methods and to the six prognostic profiles

**Prognostic profiles**	**Method 1**		**Method 2**	
	**Number of pairs**	**Number of final events**	**Number of pairs**	**Number of final events**
		**Pregnancy/nonpregnancy group**	**Imperfect/perfect**	**Pregnancy/nonpregnancy group**
*RH*−and good*cPP*	7	2/2	1/6	2/0
*RH*−and bad*cPP*	16	4/2	1/15	4/2
*RH*+not treated and good*cPP*	12	4/2	1/11	4/1
*RH*+not treated and bad*cPP*	19	5/5	0/19	5/7
*RH*+treated and good*cPP*	5	0/1	2/3	0/1
*RH*+treated and bad*cPP*	9	1/4	0/9	1/2

The number of imperfect pairs is also given for Method 2.

The *HP* model applied with Method 1 yielded the following estimations: $\exp\left(\gamma \right)=0.90$, *CI*_95*%*_[ 0.36−2.21], not significant. Kranick *et al.*[5] and Veletgas *et al.*[4] concluded likewise as did Azim *et al.*[3], but the latter stratified their analysis on estrogen status. Other authors [7,10] found a protective role of pregnancy. All these authors used the *HP* model with Method 1 adjusted for the other known prognostic factors. In view of our simulation results, we believe that this estimation was underestimated and that the models should be used on censored correlated data created with Method 2. Regarding the latter, the *HP* model yielded a larger value than with Method 1 but was still not significant ($\exp\left(\gamma \right)=2.00$, *CI*_95*%*_[ 0.75−5.33]). In view of our simulations, we conclude that $\bar{\text{HR}}\left(t\right)$ was widely underestimated with *HP* using Method 1 and only slightly underestimated with *HP* using Method 2. The increasing value of $\bar{\text{HR}}\left(t\right)$ estimated by *HP* between the two matching methods was not surprising. Method 2 and the *LWA* model, with or without adjusting for matching covariates (*LWA*_*u*_ and *LWA*_*a*_), yielded similar and not statistically significant results: $\exp\left(\gamma \right)=1.22$, *CI*_95*%*_[ 0.61−2.42] and $\exp\left(\gamma \right)=1.24$, *CI*_95*%*_[ 0.62−2.45], respectively. The difference in $\bar{\text{HR}}\left(t\right)$ values estimated by the *HP* and *LWA*_*u*_ models was not statistically significant. In such a context, we would like to estimate the proper effect of the subsequent pregnancy. It is more relevant to estimate ${\bar{\text{HR}}}_{a}\left(t\right)$ than $\bar{\text{HR}}\left(t\right)$. Even if *LWA*_*i*_ showed no significant interaction between the matching covariates and the pregnancy, because of the biological and clinical assumptions, we estimated the effect of pregnancy according to the six PP defined above using the *LWA*_*i*_ model. Table 5 presents $\exp\left(\gamma \right)$ estimations according to the models and their 95*%* confidence intervals.

**Table 5:** exp(γ)** estimations and their 95% confidence interval according to the *HP * and *LWA * models and to the matching methods, with the p-value of the Wald’s Test**

**Models**	**Method 1**	**Method 2**
Holt and Prentice model	$$\begin{array}{c} \exp\left(\gamma \right)=0.90\\ {\text{CI}}_{95}=\;[\;0.36-2.21]\\ p-\text{value}=0.82\\ \text{se}=0.46 \end{array}$$	$$\begin{array}{c} \exp\left(\gamma \right)=2.00\\ {\text{CI}}_{95}=\;[\;0.75-5.33]\\ p-\text{value}=0.17\\ \text{se}=0.50 \end{array}$$
Lee, Wei and Amato model		
*LWA*_*u*_		$$\begin{array}{c} \exp\left(\gamma \right)=1.22\\ {\text{CI}}_{95}=\;[\;0.61-2.42]\\ p-\text{value}=0.58\\ \text{se}=0.35 \end{array}$$
*LWA*_*a*_		$$\begin{array}{c} \;\\ \exp\left(\gamma \right)=1.24\\ {\text{CI}}_{95}=\;[\;0.62-2.45]\\ p-\text{value}=0.54\\ \text{se}=0.35 \end{array}$$
*LWA*_*i*_:*RH*− and good cPP		$$\begin{array}{c} \;\\ \exp\left(\gamma \right)=5.70\\ {\text{CI}}_{95}=\;[\;0.80-40.50]\\ p-\text{value}=0.08\\ \text{se}=0.98 \end{array}$$
*LWA*_*i*_:*RH*− and poor cPP		$$\begin{array}{c} \;\\ \exp\left(\gamma \right)=1.77\\ {\text{CI}}_{95}=\;[\;0.34-9.24]\\ p-\text{value}=0.49\\ \text{se}=0.83 \end{array}$$
*LWA*_*i*_:*RH*+ not treated and good cPP		$$\begin{array}{c} \;\\ \exp\left(\gamma \right)=2.87\\ {\text{CI}}_{95}=\;[\;0.63-13.10]\\ p-\text{value}=0.17\\ \text{se}=0.76 \end{array}$$
*LWA*_*i*_:*RH*+ not treated and poor cPP		$$\begin{array}{c} \;\\ \exp\left(\gamma \right)=0.89\\ {\text{CI}}_{95}=\;[\;0.30-2.69]\\ p-\text{value}=0.83\\ \text{se}=0.55 \end{array}$$
*LWA*_*i*_:*RH*+ treated and good cPP		$$\begin{array}{c} \;\\ \exp\left(\gamma \right)=0.95\\ {\text{CI}}_{95}=\;[\;0.07-13.66]\\ p-\text{value}=0.97\\ \text{se}=1.33 \end{array}$$
*LWA*_*i*_:*RH*+ treated and poor cPP		$$\begin{array}{c} \;\\ \exp\left(\gamma \right)=0.30\\ {\text{CI}}_{95}=\;[\;0.04-2.42]\\ p-\text{value}=0.25\\ \text{se}=1.05 \end{array}$$

The estimation of the pregnancy effect standard error (*se*) by the *HP* and *LWA* models are given.

The apparent protective role of pregnancy for the theoretically less favorable prognostic profile “ *RH*+ (treated or not) and poor *cPP*” and its increasing role in risk for the theoretically best prognostic profile “ *RH*− and good *cPP*” were surprising. There was no statistical significance, but it could be because of a lack of power of the combination Method 2 - *LWA*_*i*_.

## Discussion

In our context of exposure occurring over time, we focused on two matching methods: Method 1, commonly used in the literature [3]-[5,7,10], and Method 2, our new approach. Method 1 composes the pairs in an *a posteriori* way where the pairs are in fact considered to be known at diagnostic time *t*=0. Method 2 creates pairs in “real-time”. Such matching designs create independence between pairs but dependence between the subjects of the same pair, and specific analytical methods exist for such a situation of correlated censored data. In our work we studied two semi-parametric models allowing for the stratified Holt and Prentice model [16] (*HP*) and the Lee, Wei and Amato model [20] (*LWA*). To estimate the average exposure effect *HR*(*t*) and unlike the *LWA*, the *HP* did not make it possible to take into account either the matching covariates or the possible interaction between the matching covariates and the exposure.

The aim of this study was to analyze the *HP* and *LWA* models using the two matching methods in order to propose the most efficient Method - Model combination to estimate and test the prognostic exposure effect *HR*(*t*) estimated through the models by $\exp\left(\gamma \left(t\right)\right)$.

In view of our simulations, the relative difference in the number of pairs between Method 1 and Method 2 depends on the ratio $\lambda_{12}\left(t\right)/\lambda_{13}\left(t\right)$ and on the censoring percent. Compared to Method 1, the number of pairs was equal or larger with Method 2. In terms of bias and *RMSE*, Method 2 is more relevant than Method 1 to design the pairs, and *LWA*_*a*_ is the best model for all the situations except when there is an interaction between the covariates and the exposure (***β***_23_≠ ***β***_13_), for which *LWA*_*i*_ is more appropriate even if the *HR*_*i*_(*t*) estimations are not uniformly unbiased.

In our sample data, we applied Method 1 and *HP* to compare our results with those in the literature [3]-[5,7,10]. According to the simulation results, we applied Method 2 with *HP* and *LWA*. With both matching methods, we obtained an equal number of pairs (the maximum available) but not the same ones. *HP* used with Method 1 gave the smallest estimation of $\bar{\text{HR}}\left(t\right)$. $\bar{\text{HR}}\left(t\right)$ estimations were not statistically different from 1 with *HP*, *LWA*_*u*_ and *LWA*_*a*_. According to biological and clinical hypothesis, we assumed that the effect of exposure was different according to the six prognostic profiles so we used the *LWA*_*i*_ model. However, we did not conclude that pregnancy had neither a protective effect nor an adverse effect on progression disease, even for women with positive hormonal receptors and poor clinical profile at diagnosis. Estimating $\bar{\text{HR}}\left(t\right)$ by prognostic profiles using the combination Method 2 - *LWA*_*i*_, showed a different and opposite effect of exposure in the six health profiles, but none was statistically significant. The sample size of the profiles was small and a few events occurred inside each profile, probably leading to a lack of power of the combination Method 2 - *LWA*_*i*_.

Adjusting for the matching covariates and for their possible interaction with exposure could be very interesting to interpret the effect of exposure *HR*(*t*). However, it could be more difficult with real data, especially in the event of multiple prognostic profiles. The sample had to be large enough to enable us to assess the performances of the models according to the two matching methods, especially *LWA*_*a*_ and *LWA*_*i*_, which required a large number of pairs. The erroneous estimation with *LWA*_*i*_ in some extreme profiles could partially be explained as follows: in the “imperfect” pairs, we artificially prevent the non-exposed subject from having a final event, because she will first become an exposed subject of another pair. Maybe she will undergo the final event but as an exposed subject and in another pair. Then, when the proportion of imperfect pairs is large, *HR*_*i*_(*t*) could be overestimated. Moreover the larger the ratio $\lambda_{12}\left(t\right)\lambda_{13}\left(t\right)$, the larger the number of imperfect pairs, and the higher the overestimation of *HR*_*i*_(*t*) and the faster its appearance. Owing to the values of the *β*_*u* *v* *k*_ triplets chosen in the particular configuration presented above, the subjects of the three profiles $Z^{{}^{\prime }}\in \left\{\left(0,0,0\right),\left(1,0,0\right),(0,1,0)\right\}$ experienced more exposure before any events, and simultaneously fewer events (before or after exposure), than the other PP. In these three profiles, the number of events in the exposed and in the non-exposed individuals is quite low, suggesting a possible lack of accuracy in the estimation of $\bar{\text{HR}}\left(t\right)$. All the models needed enough pairs and enough final events to fit: the larger the number of imperfect pairs and the smaller the number of events, the worse the accuracy of the estimation of *HR*_*i*_(*t*). In a future study, we will explore more deeply this problem of bias related to the percent of imperfect pairs in the sample.

Technically, whatever the matching method used, *HP* censored the pair when one of its subjects was censored or experienced the final event, whereas the *LWA* did not. The latter leaves each subject to be followed up to her censoring or final event, whatever the outcome of the matched subject. Concerning the management of the imperfect pair obtained with Method 2, the non-exposed subject *i* of pair *P*_*j*_ was censored when she became the exposed subject *i* of another pair *P*_*i*_ at the time $t_{E_{i}}$; by construction, as said before, her exposure occurred before any other events. For *LWA*, an alternative would be to censor the pair *P*_*j*_. Another alternative, whatever the model, would be to propose another non-exposed (perfect or imperfect) subject to the exposed subject of pair *P*_*j*_, who was now single in her pair. This issue deserves more investigation.

Using time intervals to estimate the effect of exposure as it changes over time was maybe not the most pertinent method, because it required many parameters. Therefore, to improve fitting, we intend to apply splines to fit the effect of exposure as it changes over time. Indeed, the present study sought to compare results of two known models devoted to censored correlated data, and the well-known frailty model was set aside because it requires the structure of correlation within the pairs to be specified. The next step would be to compare the *HP*, *LWA* and the frailty model using Method 2.

## Conclusions

In conclusion, correlated censored data designing by Method 2 seems to be the more pertinent method to create pairs when the criterion which characterizes the pair is an exposure occurring over time. It would be interesting to estimate the proper effect of the subsequent pregnancy. It is then more pertinent to estimate ${\bar{\text{HR}}}_{a}\left(t\right)$ than $\bar{\text{HR}}\left(t\right)$. Thus, *LWA*_*a*_ seems to be the best model for all the situations, except when there is an interaction between the covariates and the exposure, for which *LWA*_*i*_ is more appropriate, even if the estimations of *HR*_*i*_(*t*) are not uniformly unbiased. *LWA*_*a*_ and *LWA*_*i*_ gave a more accurate and relevant estimation of the effect of exposure in particular context, where we can reasonably suppose that the latter depends on prognostic profiles.

## Appendix A: Simulation of cohort data - Procedures and scenarios chosen

For each subject four independent times were generated: *t*_12_, *t*_23_, *t*_13_ and *C* (censoring time), according to the intensities displayed in Table 2. From these times, 2 times of interest for each subject were derived: a time to exposure *t*_12_=*t*_*E*_ and a time to final event *t*_*i*_. Two indicator variables were derived: *E*=1 if an exposure occurs, 0 otherwise and *Δ*=1 if a final event occurs, 0 otherwise. Four possible quadruplets (*t*_*E*_;*t*_*i*_;*E*;*Δ*) could be defined as follows:

$$\begin{array}{lcrr} (C;C;0;0)\text{if}C & < & \min(t_{12},t_{13})\text{,} & \\ (t_{13};t_{13};0;1)\text{if}t_{13} & \leq & \min(t_{12},C)\text{,} & \\ (t_{12};C;1;0)\text{if}t_{12} & < & \min(t_{13},C)\text{and}t_{12}+t_{23}>C, & \\ (t_{12};t_{12}+t_{23};1;1)\text{if}t_{12} & < & t_{13}\text{and}t_{12}+t_{23}\leq \mathrm{C.} & \end{array}$$

Such a design refers to an “illness-death” model with transition intensities $\lambda_{12}\left(t\right)$, $\lambda_{13}\left(t\right)$ and $\lambda_{23}\left(t\right)$ (Figure 1). All subjects were assumed to be in the initial state (state 1 or cancer diagnosis in our context) at time *t*=0. They could move to the final state (state 3 or disease progression) with a transition intensity $\lambda_{13}\left(t\right)$. They might undergo the intermediate event or exposure (state 2 or pregnancy) with intensity $\lambda_{12}\left(t\right)$, before developing any progression with intensity $\lambda_{23}\left(t\right)$. Date of entry into state 1 was chosen as time of origin for all transitions. Thus the parameter of interest *HR*(*t*) corresponded to the ratio $\lambda_{23}\left(t\right)/\lambda_{13}\left(t\right)$. However, to compute $\lambda_{23}\left(t\right)$, we took into account the left truncation phenomenon: before being at risk of an event in the transition 2→3, a subject has to wait until its exposure occurs. This delayed entry leads the set of subjects at risk in transition 2→3 to increase when an exposure occurs and to decrease when an event occurs. Thus the average *HR*(*t*) is obtained from an exact formula involving the averages of $\lambda_{13}\left(t\right)$ and $\lambda_{23}\left(t\right)$ which are computed through a numerical approximation (transformation of the time from continuous to discrete values) (See the Appendix B: Calcul of the average exposure effect not adjusted for the covariates). The average *HR*(*t*) adjusted for the different covariates was estimated empirically by using large size samples to guarantee good precision. Moreover, note that the larger the ratio $\lambda_{12}\left(t\right)/\lambda_{13}\left(t\right)$, the larger the number of exposures in the simulated cohort.

The simulation model included (i) the choice of an instantaneous baseline risk function $\lambda_{\text{uv}}\left(t,Z\right)$ for each of the three transitions u→v, (ii) the choice of the **Z** effects, $\exp\left(\beta_{\text{uvk}}\right)$, for each transition and (iii) the choice for the censoring proportion.

For (i), an instantaneous average risk function $\lambda_{\text{uv}}\left(t,Z=\bar{Z}\right)$ for each of the three transitions was simulated: either a constant risk using an exponential density function ^(1)^, a monotone risk using a Weibull density function ^(2)^ or an increasing then decreasing risk using a loglogistic density function ^(3)^. Five $\lambda_{\text{uv}}\left(t,Z=\bar{Z}\right)$ triplets were simulated in order to construct five realistic configurations of *HR*(*t*): two constant, one increasing, one decreasing and one increasing then decreasing, where *HR*(*t*) range values were clinically pertinent (between 0.5 and 4 in the whole population). Table 2 displays the $\lambda_{\text{uv}}\left(t,Z=\bar{Z}\right)$ distributions of each transition used for each of the five different configurations of *HR*(*t*).

For (ii), different *β*_*u* *v* *k*_ values for each of these five $\lambda_{\text{uv}}\left(t,Z=\bar{Z}\right)$ triplets were chosen. Negative *β*_12_ values were proposed and set at $\beta_{12}^{{}^{\prime }}=\left(-0.2,-0.4,-0.8\right)$. Only ***β***_13_ and ***β***_23_ had other possible values which were the following: (−0.2,−0.4,−0.8), (+0.2,+0.4,+0.8), (−0.1,−0.2,−0.4) and (+0.1,+0.2,+0.4). Ten *β*_*u* *v* *k*_ scenarios were performed. Given the five configurations chosen for *HR*(*t*) and the ten *β*_*u* *v* *k*_ scenarios, 50 different situations were obtained.

Finally, for (iii), these previous 50 situations were first performed without censoring. To minimize simulations time, two levels of independent uniform censoring were implemented only with the following *β*_*u* *v* *k*_ scenario: $\beta_{12}^{{}^{\prime }}=\left(-0.2,-0.4,-0.8\right)$, ***β***_13_=−***β***_12_ and ***β***_23_= ***β***_12_; and they were applied to each of the five configurations of *HR*(*t*). This yielded to 10 more situations (five *HR*(*t*) configurations with2 levels of censoring) for that *β*_*u* *v* *k*_ scenario. The maximal event time $t_{\max}$ was set at 1000. The first uniform distribution for censoring time *C* was over the interval time $\left[0;t_{\max}\right]$, and the second one over $\left[0;2t_{\max}\right]$; then the maximal censoring time was $C_{\max}=+\infty$, $t_{\max}$ or $2t_{\max}$. The overall censoring level was higher in the first censoring distribution but it also depended on the *HR*(*t*) configuration. In total we had50 situations without censoring and 10 with censoring (the same five configurations with the two levels of censoring).

For each of the 60 situations, 1000 different data sets were generated with a sample size of 2000 subjects. At *t*=0, these 2000 subjects were allocated to the eight **Z** profiles. At *t*>0, the 250 subjects of the 8 different profiles are divided up among the three transitions and change over time according to the five *HR*(*t*) configurations.

## Appendix B: Calcul of the average exposure effect not adjusted for the covariates $\bar{\text{HR}}(t)$

Density function $f_{23}\left(t\right),$ Repartition function $F_{23}\left(t\right)$ and Survival function $S_{23}\left(t\right)$ of transition 2→3 taking into account the left truncation

$$\begin{array}{lcrr} f_{23}\left(t\right) & = & \int_{0}^{t}f_{23}\left(t-u\right)f_{12}\left(u\right)S_{13}(u)\text{du}, & \\ F_{23}\left(t\right) & = & \int_{0}^{t}f_{23}\left(v\right)\text{dv}=\int_{0}^{t}\int_{0}^{v}f_{23}\left(v-u\right)f_{12}\left(u\right)S_{13}(u)\text{du}, & \end{array}$$

where *u* is the exposure occurrence time and *t* the final event time.

To obtain an useful formula we divide the time in *K* tiny intervals with $t_{0}<t_{1}<\cdots <t_{k}<\cdots <t_{K}=T_{\max}$

$$\begin{array}{lcrr} S_{23}\left(t_{k}\right) & = & \overset{k}{\underset{j=1}{\prod }}\;\left(\;\;1\;-\;\frac{f_{23}\left(t_{j}\right)\left(t_{j}-t_{j-1}\right)}{\int_{0}^{t_{j}}f_{12}\left(x\right)S_{13}(x)\text{dx}-\;\overset{j-1}{\underset{l=1}{\sum }}\left(t_{l}-t_{l-1}\right)f_{23}\left(t_{l}\right)}\;\;\right) & \\ & = & \overset{k}{\underset{j=1}{\prod }}\;\left(\;\;1\;-\;\frac{f_{23}\left(t_{j}\right)\left(t_{j}-t_{j-1}\right)}{\int_{0}^{t_{j}}f_{12}\left(x\right)S_{13}(x)\text{dx}-\;\overset{j-1}{\underset{l=1}{\sum }}\left(t_{l}-t_{l-1}\right)f_{23}\left(t_{l}\right)}\;\;\right) & \\ & = & \overset{k}{\underset{j=1}{\prod }}\left(\frac{\int_{0}^{t_{j}}f_{12}\left(x\right)S_{13}(x)\text{dx}-\overset{j}{\underset{l=1}{\sum }}\left(t_{l}-t_{l-1}\right)f_{23}\left(t_{l}\right)}{\int_{0}^{t_{j}}f_{12}\left(x\right)S_{13}(x)\text{dx}-\overset{j-1}{\underset{l=1}{\sum }}\left(t_{l}-t_{l-1}\right)f_{23}\left(t_{l}\right)}\right) & \end{array}$$

and

$$\lambda_{23}(t)=\frac{-\text{Log}\left(S_{23}\left(t_{k}\right)\right)}{\left(t_{k}-t_{k-1}\right)}.$$

The average instantaneous hazard $\bar{\lambda }\left(t\right)$ is equal to

$$\begin{array}{lcrr} \bar{\lambda }\left(t\right) & = & \frac{\underset{z}{\sum }\omega_{z}\left(t\right)\lambda \left(t\left|Z\right)\right)}{\underset{z}{\sum }\omega_{z}\left(t\right)} & \\ \omega_{z}\left(t\right) & = & \pi \left(z\right)S\left(t\left|Z\right)\right) & \\ {\bar{\omega }}_{z}\left(t\right) & = & \frac{\omega_{z}\left(t\right)}{\underset{z}{\sum }\omega_{z}\left(t\right)} & \\ \bar{\lambda }\left(t\right) & = & \underset{z}{\sum }{\bar{\omega }}_{z}\left(t\right)\lambda \left(t\left|Z\right)\right)=\underset{z}{\sum }{\bar{\omega }}_{z}\left(t\right)\lambda_{z}\left(t\right) & \\ {\bar{\omega }}_{z}\left(t\right) & = & \frac{N_{z}\left(t\right)}{\underset{z}{\sum }N_{z}\left(t\right)} & \end{array}$$

where $\lambda \left(t\left|Z\right)\right)=\lambda_{z}\left(t\right)$ is the average instantaneous hazard in the profile *z* at time *t*, $N_{z}\left(t\right)$ is the number of subject at risk in the profile *z* at time *t*. Then we can write

$$\bar{\text{HR}}(t)=\frac{{\bar{\lambda }}_{23}(t)}{{\bar{\lambda }}_{13}(t)}=\underset{z}{\sum }\frac{\frac{N_{23_{z}}\left(t\right)}{\underset{z}{\sum }N_{23_{z}}\left(t\right)}\lambda_{23_{z}}\left(t\right)}{\frac{N_{13_{z}}\left(t\right)}{\underset{z}{\sum }N_{13_{z}}\left(t\right)}\lambda_{13_{z}}\left(t\right)}.$$

The computation of $N_{z}\left(t\right)$ is the following.

In transition 1→3, a subject is at risk if it does not have any event and if it is not censored

$$N_{13_{z}}\left(t\right)=N_{13_{z}}S_{13_{z}}\left(t\right)\bar{G}\left(t\right)S_{12_{z}}\left(t\right),$$

where 

• $S_{12_{z}}(t)$ is the survival rate in the transition 1→2 for the profile *z* at time *t*,

• $S_{13_{z}}(t)$ is the survival rate in the transition1→3 for the profil *z* at time *t*,

• $N_{13_{z}}$ is the number of subject at risk in the profile *z* at time *t*=0,

• $\bar{G}\left(t\right)=1-G\left(t\right)$ with *G*(*t*) is the repartition function of the variable *C* characterizing the censoring time. It does not depend on the profile.

In transition 2→3, taking into account the left truncation, a subject is at risk of event in this transition, if it entered in the transition and if it had neither an event nor being censored.

$N_{23_{z}}$ could be expressed as

$$N_{23_{z}}\left(t\right)=\left[E_{23_{z}}\left(t\right)-D_{23_{z}}\left(t\right)-C_{23_{z}}\left(t\right)\right]N_{23_{z}}$$

where 

• $E_{23_{z}}\left(t\right):$ the number of subjects which entered in the transition 2→3 in the time interval [ 0;*t*],

• $D_{23_{z}}\left(t\right):$ the subjects which undergo the final event in the time interval [ 0;*t*],

• $C_{23_{z}}\left(t\right):$ the censored subject in the time interval [ 0;*t*],

• $N_{23_{z}}$ is the number of subject at risk in the profile *z* at time *t*=0.

Then

$$\begin{array}{lcrr} \;N_{23_{z}}\;\left(t\right)\;\; & = & \;\;\left[E_{23_{z}}\left(t\right)-D_{23_{z}}\left(t\right)-C_{23_{z}}\left(t\right)\right]N_{23_{z}} & \\ & = & \;\;\left[\;\;\;\begin{array}{l} \int_{0}^{t}\bar{G}\left(x\right)\lambda_{12_{z}}\left(x\right)S_{12_{z}}\left(x\right)S_{13_{z}}\left(x\right)\text{dx}\\ -\int_{0}^{t}\bar{G}\;\left(x\right)\;\int_{0}^{x}\lambda_{12_{z}}\;\left(v\right)\;S_{12_{z}}\;\left(v\right)\;S_{13_{z}}\;\left(v\right)\;f_{23_{z}}\;\left(x\;-\;v\right)\text{dvdx}\\ -\int_{0}^{t}g\;\left(x\right)\;\int_{0}^{x}\lambda_{12_{z}}\;\left(v\right)\;S_{12_{z}}\;\left(v\right)\;S_{13_{z}}\;\left(v\right)\;S_{23_{z}}\;\left(x\;-\;v\right)\text{dvdx} \end{array}\;\right]\;N_{23_{z}}, & \end{array}$$

where 

• $\lambda_{12_{z}}(t)$ is the instantaneous hasard function of transition 1→2 for the profil *z* at time *t*,

• $g\left(t\right)$ is the density function of the variable *C* characterizing the censored time. It does not depend on the profile.

## Appendix C: Empirical estimation of the average exposure effect adjusted for the covariates ${\bar{\text{HR}}}_{a}(t)$

Let’s consider *L* intervals *I*_*l*_ (*l*=1 to *L*) inside the study interval [ 0;*T*_*m* *a* *x*_], where all the *i* subjects are censored at time *T*_*m* *a* *x*_: $I_{l}=]a_{l-1};a_{l}]:l=1,\cdots \;,L$ with

$$a_{0}=0<a_{1}<a_{2}<\cdots <a_{L}=T_{\max}.$$

Inside each *I*_*l*_, the exposure variable *E*_*l*_(*t*) is as follows:

$$E_{l}\left(t\right)=\left\{\begin{array}{ll} 1 & \text{if}t\geq t_{\text{Ei}}\text{and}t\in I_{l}\\ 0 & \text{otherwise}. \end{array}\right)$$

Then we define: $E\left(t\right)=\overset{L}{\underset{l=1}{\sum }}E_{l}\left(t\right).$

Our model of simulation obtained from an “illness-death” model is as follows:

$$\begin{array}{ll} \;\lambda \left(t,Z,E\right) & =\lambda_{0}\left(t\right)\exp\left(\overset{L}{\underset{l=1}{\sum }}\gamma_{l}E_{l}\left(t\right)+\beta_{1}Z_{1}+\beta_{2}Z_{2}\right)\\ & \;\left(+\;\beta_{3}Z_{3}+\left(\alpha_{1}Z_{1}+\alpha_{2}Z_{2}+\alpha_{3}Z_{3}\right)E\left(t\right)\right), \end{array}$$

where *λ*(*t*,**Z**,*E*) is the instantaneous risk function of state 3 occurrence according to the exposure *E* and the covariates *Z*_*k*_ (*k*=1*to* 3).

From this model, we are able to calculate *HR*_*i*_(*t*), *i.e.* the *HR*(*t*) for each profile (*Z*_1_, *Z*_2_, *Z*_3_):

$${\text{HR}}_{i}\left(t\right)=\frac{\lambda \left(t,Z,E=1\right)}{\lambda \left(t,Z,E=0\right)}.$$

We approximate *HR*_*a*_(*t*) by

$${\bar{\text{HR}}}_{a}\left(t\right)=\exp\left(\overset{L}{\underset{l=1}{\sum }}\gamma_{l}E_{l}\left(t\right)\right),$$

where the *γ*_*l*_ are obtained from

$$\;\lambda \left(t,Z,E\right)=\lambda_{0}\left(t\right)\exp\left(\overset{L}{\underset{l=1}{\sum }}\gamma_{l}E_{l}\left(t\right)+\beta_{1}Z_{1}+\beta_{2}Z_{2}+\beta_{3}Z_{3}\right).$$

We approximate *HR*(*t*) by

$$\bar{\text{HR}}\left(t\right)=\exp\left(\overset{L}{\underset{l=1}{\sum }}\gamma_{l}E_{l}\left(t\right)\right),$$

where the *γ*_*l*_ are obtained from

$$\lambda \left(t,Z,E\right)=\lambda_{0}\left(t\right)\exp\left(\overset{L}{\underset{l=1}{\sum }}\gamma_{l}E_{l}\left(t\right)\right).$$

For ${\bar{\text{HR}}}_{a}(t)$, the estimation of *γ*_*l*_ is adjusted for the *β*_*k*_, whereas for $\bar{\text{HR}}(t)$ it is not.

The approximation of *HR*_*i*_(*t*), ${\bar{\text{HR}}}_{a}(t)$ and $\bar{\text{HR}}(t)$ is especially relevant when *L* and the total number of patients are large, in order to have enough exposure and events inside the transitions 2→3 and 1→3.

The approximations of ${\text{HR}}_{i}\left(t\right)$ and $\bar{\text{HR}}\left(t\right)$ could be compared to the theoretical values (See Appendix B: Calcul of the average exposure effect not adjusted for the covariates), not of ${\bar{\text{HR}}}_{a}(t)$.

To be sure of the approximation of ${\bar{\text{HR}}}_{a}(t),$ we simulated data from a Cox proportional hazards model with a time-dependent covariate representing exposure. As we obtained directly the effect of the exposure we were able to certify that our approximation’s method was correct.

## Competing interests

The authors declare that they have no competing interest.

## Authors’ contributions

AS, CG, PTB and YDR conceived the research question. AS and CG conducted the literature review. AS drafted the manuscript, contributed to the interpretation of all the simulations results, and analyzed and interpreted the real data on pregnancy and breast cancer. PTB and YDR revised the manuscript and supervised AS at all stages. YDR oversaw the design, programmed and ran the simulation study. All authors read and approved the final manuscript.

## Pre-publication history

The pre-publication history for this paper can be accessed here:

http://www.biomedcentral.com/1471-2288/14/83/prepub
